# Supplementary material for: Table 2 Fallacy in Descriptive Epidemiology: Bringing Machine Learning to the Table
Source: Int J Environ Res Public Health. 2023 Jun 21;20(13):6194. doi: 10.3390/ijerph20136194 (PMC10340623; doi:10.3390/ijerph20136194)

Supplementary Materials

Table S1. Data dictionary for all variables included in the 4 machine learning models

	**Definition**	**Levels**	
**Outcome**			
Alcohol abuse	Past 12 month alcohol abuse, defined as use of alcohol more than once a week AND have had 5 or more drinks in one occasion at least once	0 = none or occasional use, 1 = frequent alcohol abuse	
**Socio-demographic**			
age	Age	Continuous (from 15 -29 years)	
province	Canadian Province	7= Ontario, 9= Quebec	
curr_orient2	Current sexual orientation	0= asexual, 1 = heteroflexible / straight or heterosexual / questioning or other, 2 = bisexual, 3 =	

		gay, 4 = lesbian, 5 = pansexual, 6 = queer / Two spirit	
gender	Current gender identity	0= cisgender man, 1= cisgender woman, 2 = trans man, 3 = trans woman, 4 = genderfluid / genderqueer / gender non-binary / gender non-confirming / Two-spirit	
residence	How long living in Canada	1 = 2 years or less, 2 = 3 to 5 years, 3 = 6 to 10 years, 4 = 11 to 20 years, 5 = Canadian born	
education	Highest level of education completed	0= high school or below, 1= college or diploma or similar, 2= university degree	
employ	Employment status	0= unemployed (including student, working without pay and others), 1= employed	

		(including self-employed); 2 = student only	
house_income	Combined income of everyone in the house for the past year	0 = Less than $15,000<br>1 = $15,000 to $29,999<br>2 = $30,000 to $59,999<br>3 = $60,000 to $79,999<br>4 = $80,000 to $100,000<br>5 = More than $100,000	
ind_income	Individual income for the past year	0 = Less than $15,000<br>1 = $15,000 to $29,999<br>2 = $30,000 to $59,999<br>3 = $60,000 to $79,999<br>4 = $80,000 or more	
Rural city	What best describes the environment you live in?	1 = Large urban center (100,000+)<br>2 = Medium city / town (30,000 – 99,000)	

		3 = Small city / town (1,000 – 29,999) 4 = Rural area (< 1,000)	
where_live	Current living situation or accommodation	0= not in own place (including shelter, supportive housing or homeless), 1= own place or rent own place with roommate or partner, 2 = live with parents / guardian	
Trans status	Transgender identity	0 = No; 1 = Yes	
intersex	Intersex	0 = No; 1 = Yes	
POC	Identify as a person of colour	0 = No; 1 = Yes	
**General health**			
gen_health	Health status ratings	1 – 5 (poor to excellent)	
fitness1	Past 7 day, sports or physical activities for 10 continuous mins.	0= no, 1= yes	

treat_comorbidities	Diagnosed with comorbid conditions: chronic lung disease, asthma, chronic pain, diabetes, heart disease, lung cancer, other cancers	0= no, 1= yes. (Note: nobody has cancer, so we did not use it) [c]	
disability	Living with condition: intellectual disability, learning disability, Autism, Asperger's or neurodiverse spectrum, difficulty in seeing, hearing, walking, using hands or fingers, doing physical activities, chronic pain	0= no, 1= yes	
**Mental health**			
mental_health	Mental health ratings	1 – 5 (poor to excellent)	

stresslife	Stress levels in most days	1 – 5 (extremely stressful to not stressful)	
con_eating	diagnosed mental health condition: anorexia or bulimia	0= no, 1= yes	
con_anxiety	diagnosed mental health condition: anxiety disorder	0= no, 1= yes	
con_ADD	diagnosed mental health condition: ADD	0= no, 1= yes	
con_ADHD	diagnosed mental health condition: ADHD	0= no, 1= yes	
con_bipolar	diagnosed mental health condition: bipolar disorder	0= no, 1= yes	
con_depression	diagnosed mental health condition: depression disorder	0= no, 1= yes	

con_OCD	diagnosed mental health condition: OCD	0= no, 1= yes	
con_panic	diagnosed mental health condition: panic disorder	0= no, 1= yes	
con_PTSD	diagnosed mental health condition: PTSD	0= no, 1= yes	
con_others	diagnosed mental health condition: others (including dysthymia, mania, phobia, psychosis, and schizophrenia)	0= no, 1= yes	
suicidal	Past 12 month suicidal thought	1 = never, 2 = less than once a month, 3 = 1 to 3 times per month, 4 = 1 to 6 times per week, 5 = every day	

Seek_help	Wanted to talk or seek help with a professional	0 = No, 1 = Yes	
Receive_help	Delay the mental health care need	0 = No, 1 = Yes, 2 = did not seek help	
**Smoking related behaviours**			
curr_smoke	Current smoking frequency	0= non-smoker, 1= daily or almost daily, 2= less than daily but at least weekly, 3 = less than weekly but at least monthly, 4 = less than monthly	
use_cigar	Past 30 day use of cigar or cigarillos	0= no, 1= yes	
use_wp	Past 30 day use of waterpipe (e.g., hookah, sheesha)	0= no, 1= yes	
use_smokeless	Past 30 day use of smokeless tobacco (e.g., snus.chew)	0= no, 1= yes	

curr_vape	Current vaping frequency	0= never vaper, 1= current vaper, 2= ever vaper	
**Substance use**			
poppers	Past 12 month use of poppers/amyl	0 = no, 1= yes	
crystal_meth	Past 12 month use of crystal meth/Tina	0 = no, 1= yes	
crack	Past 12 month use of crack, free base	0 = no, 1= yes	
cocaine	Past 12 month use of cocaine	0 = no, 1= yes	
heroin	Past 12 month use of heroin(smack)	0 = no, 1= yes	
pres_opioids	Past 12 month use of prescription opioids (e.g., Percocet, Dialudid, OxyContin)	0 = no, 1= yes	

fentanyl	Past 12 month use of Fentanyl	0 = no, 1= yes	
Psychedelic use	Past 12 month use of psychedellic	0 = No; 1 = Yes	
GHB	Past 12 month use of GHB/ "G"	0 = no, 1= yes	
tranquilizers	Past 12 month use of tranquilizers or benzos (e.g., Valium, Xanax)	0 = no, 1= yes	
cannabis	Past 12 month use of cannabis	0 = none, 1 = frequent (more than once a week), 2 = sometimes (less than once a week)	
drug_others	Past 12 month use of other drugs	0 = no, 1= yes	
**Composite scores: made following imputation, all scores were standardized into standard Normal distribution**			
cen_identity	Overall identity centrality score (e.g.: agreement with	Range 4-20 (strongly disagree to strongly agree); standardized to	

	statements such as “My sexual orientation/gender identity is a central part of my identity”)	standard normal with mean 0 and SD 1	
outness	Average openness score regarding sexual orientation with family, friends and other people	Range 0-7 (“definitely doesn’t know” to “definitely knows, talk about”); standardized to standard normal with mean 0 and SD 1	
connect_com	Connection to the community (e.g.: “You feel you're a part of your community”)	Range 7-35 (“strongly disagree” to “strongly agree”; standardized to standard normal with mean 0 and SD 1	
per_stigma	Overall perceived stigma score (e.g. Heard that being LGBTQI2S+ is not normal?)	Range 5-25 (“never” to “many times”); standardized to standard normal with mean 0 and SD 1	

en_stigma	Overall enacted stigma score (e.g. Been hit or beaten up for being LGBTQI2S+?)	Range 8-40 (“never” to “many times”); standardized to standard normal with mean 0 and SD 1	
phobia	Overall internalized homophobia score (I am comfortable with people knowing that I identify as LGBTQI2S+)	Range 13-53 (“Strongly disagree” to “strongly agree”; standardized to standard normal with mean 0 and SD 1	
ace	Overall adverse childhood experiences (ACEs) score (e.g. “Did you live with anyone who was depressed, mentally ill or suicidal?” Or “How often did anyone at least five years older than you or an adult	Range 0-12 (for binary items: 1 = Yes; 0 = No; frequency items: 1 = more than once or once; 0 = never) standardized to standard normal with mean 0 and SD 1	

	ever force you to have sex?”)		
Cesd_score	Sum of CESD score at baseline	Continuous score (7 to 28); standardized to standard normal with mean 0 and SD 1	

Table S2. Top ten variables with the largest coefficients from a traditional logistic regression method

Variable	Coefficient estimate	95% confidence interval
curr_orient23	9.47	(-726.53, 745.48)
covid1	0.95	(0.07, 1.82)
covid2	-13.60	(-722.40, 749.59)
covid3	-13.75	(-722.25, 749.75)
covid4	-13.34	(-722.66, 749.33)
plan_quit3	-13.48	(-722.52, 749.48)
cannabis1	-13.75	(-722.25, 749.74)
cannabis2	1.24	(0.84, 1.64)
cocaine1	1.28	(0.93, 1.62)
GHB1	1.02	(0.50, 1.53)

Figure S1. Top Ten Interactions Identified from each machine learning model

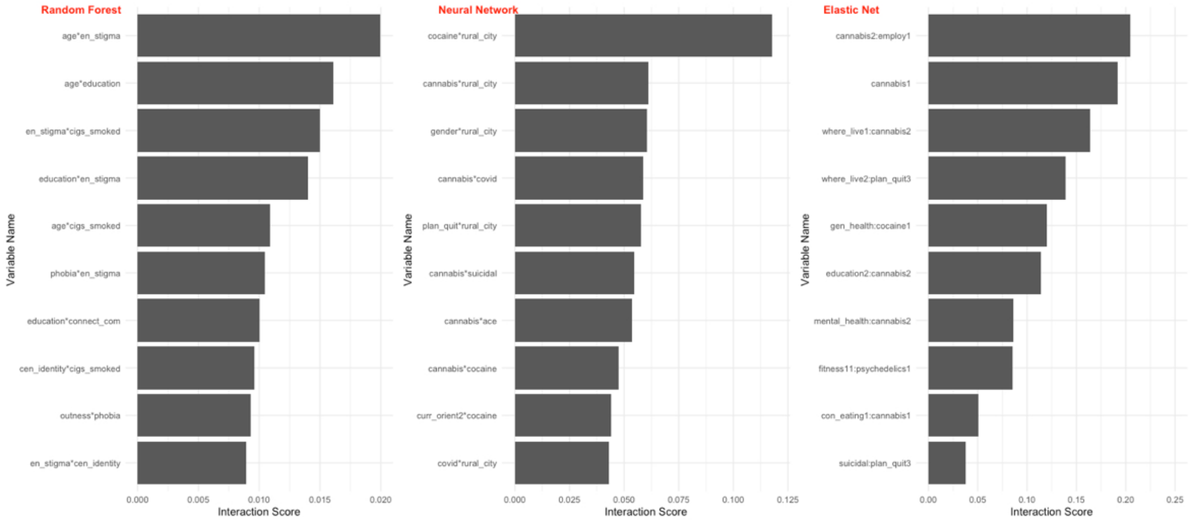

Supplement: Supplementary file 1 [file ijerph-20-06194-s001.zip › ijerph-2426505-SI.pdf]
